# Distal Femoral Replacement as a Primary Treatment Method for Distal Femoral Fractures in the Elderly

**DOI:** 10.7759/cureus.18752

**Published:** 2021-10-13

**Authors:** Ifeanyi K Onubogu, Sanjana Relwani, Urpinder S Grewal, Jagmeet S Bhamra, Kumar Gaddam Reddy, Baljinder S Dhinsa

**Affiliations:** 1 Trauma and Orthopaedics, William Harvey Hospital, Ashford, GBR; 2 Trauma and Orthopaedics, Barts and The London School of Medicine and Dentistry, London, GBR; 3 Trauma and Orthopaedics, Medway Maritime Hospital, Medway, GBR; 4 Trauma and Orthopaedics, Wexham Park Hospital, Slough, GBR

**Keywords:** fragility fractures, best practice tariff, lower extremity trauma, distal femoral replacement, distal femur fracture

## Abstract

Distal femoral fractures account for 3-6% of all femoral fractures with a similar demographic as patients suffering from proximal femoral fractures. The mortality risk can be high in such injuries, which has prompted NHS England to extend the scope of the Best Practice Tariff to include all fragility fractures of the femur. Poor bone quality, intra-articular extension, and significant comminution can make these fractures difficult to manage with fixation techniques, while early mobilisation is a key outcome in the treatment of this injury.

In this study, a comprehensive literature search was performed based on keywords, and abstracts were reviewed to identify relevant articles. The following factors were analysed: time to surgery, time to full weight-bearing, the average hospital stay, post-operative mobility status, and complications.

A total of 233 abstracts were identified using the pre-determined search criteria, and, subsequently, articles were excluded following author review. A total of 10 relevant articles were included in this review, with five used for review and comparison between distal femoral replacement (DFR) and fixation. This resulted in a sample of 200 patients treated with DFR with over 87% ambulatory at follow-up and a re-operation rate of 13.3% compared to 78% and 13.5%, respectively, in those treated with open reduction internal fixation (ORIF) procedure.

Despite a limited pool of evidence, the literature suggests that DFR offers an option that potentially allows immediate weight-bearing and leaves most patients ambulatory at follow-up. Although DFR is more costly than other operative techniques, it avoids complications associated with fixation such as non-union and can reduce the risk of further surgery through direct complications or a need for delayed arthroplasty, which is deemed more complex secondary to fixation. Early mobilisation is a key step in reducing morbidity and mortality among this cohort of patients, and a procedure such as DFR should be more widely considered to help achieve this outcome.

## Introduction and background

NHS England extended the scope of the Best Practice Tariff (BPT) for fragility hip fractures to include fractures of the femoral shaft and distal femur [1]. This change occurred in April 2020 and ensured that patients with distal femur fractures were given the same holistic care as those with proximal femur fractures.

Distal femoral fractures account for 3-6% of all femoral fractures and have a similar demographic as patients suffering from proximal femoral fractures, with an average age of 82.4 years and 83.7 years, respectively [2]. Hence, the comorbidity profile is similar, and the 30-day and one-year risk of mortality can be high in such injuries. In the elderly, these fractures are predominantly caused by low energy trauma resulting in fractures within the region extending from the metaphyseal-diaphyseal junction to the articular surface. Poor bone quality, intra-articular extension, and level of comminution can make it difficult to achieve adequate reduction and fixation in these fractures.

Treatment options vary from a conservative approach to numerous surgical options, which include intramedullary nailing, plating techniques via open reduction internal fixation (ORIF), and distal femoral replacement (DFR) [3]. The choice of treatment depends on several factors such as the presence of viable bone stock to achieve predictable fixation or concomitant knee arthritis [3]. Overall, the main goal for the treatment of these fractures is to restore distal femoral alignment to maintain function and mobility [4].

This paper aims to review the use of DFR as a primary treatment of choice for elderly patients with such injuries and compare it to locked plating techniques as an established method of fixation. The primary outcome compares weight-bearing status at the one-year follow-up, with secondary outcomes comparing time to surgery, complications, re-operation rate, length of stay, cost, and mortality.

Current management

There are numerous available options for the definitive treatment of distal femur fractures, including casting or use of hinged knee braces as conservative options, as well as intramedullary nailing and plating via ORIF as surgical options. External fixation can be used definitively in cases of severe open fractures or when the patient is unfit for further surgical treatment [4]. The overall goal for the treatment of fragility fractures is to allow early mobilisation to prevent complications related to prolonged immobility such as pneumonia, muscle atrophy, and venous thromboembolism.

Non-operative interventions include the use of casts and splints to immobilise the knee joint, where there is a stable or minimally displaced fracture configuration. This would negate the risks associated with surgery; however, it would require prolonged joint immobilisation and high levels of patient compliance to achieve a good outcome [5].

Regarding surgical intervention for distal femoral fractures in the elderly, locked plating and intramedullary nailing are the mainstays of current treatment. They offer stable fixation in good quality bone providing good resistance to inherent deforming forces [4]. However, the use of locked plates can lead to an increased risk of complications related to the rigidity of the plate and associated stiffness of the construct. Minimally invasive percutaneous osteosynthesis offers reduced morbidity associated with the standard exposure. The indications for selecting fixation include comminuted, intra-articular fractures deemed to be amenable for fixation, with specific consideration given to sufficient bone stock for permitting predictable fracture fixation [6]. Intramedullary nailing can provide good fracture stability with a minimally invasive approach. This technique has been used predominantly for fracture patterns that do not extend into the articular surface; however, advancements in technology and newer implants, such as multiple distal screw options, allow greater articular reconstruction in simple intra-articular fractures [4]. Complications with this technique include loss of distal fixation which can lead to penetration of the knee joint on axial loading.

DFR offers arthroplasty as an alternative surgical option that allows early mobilisation post-operatively and reduces the risk of complications associated with fixation. Indications for use are similar to those for fixation but highlight significant comminution at the fracture site, pre-operative pain or concomitant joint arthritis, or inadequate bone stock with osteoporosis [3]. Although there is limited evidence regarding the use of DFR as a treatment option, it may be of benefit in a select group of patients.

Distal femoral fractures are classified using the AO Foundation/Orthopaedic Trauma Association (AO/OTA) classification for metaphyseal fractures based on anteroposterior (AP) and lateral radiographs at the time of injury. These can be used to aid decision-making concerning whether to fix or replace. AO 33 type B2, B3, C2, and C3 fractures can be considered for DFR [7].

Numerous implants have been used across the literature, including Stryker’s Global Modular Replacement System (Stryker Corporation, Kalamazoo, MI, USA) and Zimmer Biomet’s Orthopaedic Salvage System (OSS) (Zimmer Biomet, Warsaw, IN, USA) with ImplantCast’s Distal Femoral Replacement (ImplantCast, Hamburg, Germany) also being used more recently. The general design of these implants is based on a modular rotating hinge knee device, with long-stemmed tibial and femoral components. Although there is no evidence to prove the superiority of one design over the others, Zimmer Biomet’s OSS has been preferred by some surgeons due to its minimum required bone resection depth of 3-5 cm [7,8]. This is lower than various other implants which require at least 7 cm as the minimum bone resection [7]. The aforementioned implants are predominantly tumour prostheses and require greater bone resection. The Link Endo-Model® (Link Orthopaedics, Hamburg, Germany) rotational hinge knee, on the other hand, is an implant that does not require full resection of condylar bone as a prosthesis that is used in complex primary and revision arthroplasty surgery. Its use in the trauma setting, however, has undergone minimal research to warrant its regular use [9]. Implants can be cemented or uncemented; however, the tendency is to cement the implants in situ, with osteoporotic bone being commonplace in the patient demographic affected by these injuries [9].

## Review

Methodology

A comprehensive literature search was performed on June 25, 2019, based on keywords, and abstracts were reviewed to identify relevant articles. We included all studies that described the use of DFR and ORIF for the treatment of distal femur fractures. Single patient case reports were excluded from this review.

The databases used to perform the search included EMBASE and Medline. The terms used for searching included “Distal femoral fracture,” “Replacement,” “Fixation,” “Repair,” and “Elderly.” The databases were searched from January 2010 until June 25, 2019.

**Figure 1:**
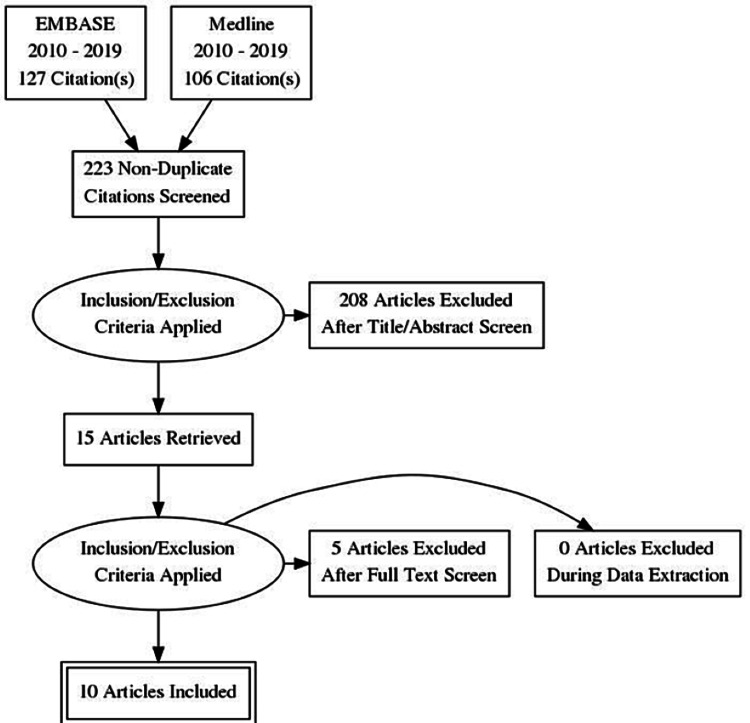
Flowchart showing the detailed article selection process.

The abstracts were reviewed independently by two authors (IO and SR). Articles that did not include elderly patients or fractures around the distal femur were excluded, along with non-English-language articles. Inclusion criteria included articles describing fixation or replacement of distal femur fractures, or those describing and comparing the two surgical options. Abstracts that were disputed for inclusion between the two authors were discussed and provisionally included for full-text review following discussion with the supervising author (BD). Following the full-text review, a total of 10 relevant articles were included in this review (Figure 1), with one article obtained from the reference review. The following factors were analysed: time to surgery, time to full weight-bearing, average hospital stay, post-operative mobility status, complications, and mortality.

Results

A total of 223 abstracts were identified using the stated search criteria. After applying exclusion criteria, 10 relevant articles were included in this review, with five used to review and compare DFR and fixation (Figure 1). The highest-level evidence provided and the majority of articles analysed were level IV evidence, such as case-control or cohort studies. This produced a sample of 200 patients treated with DFR, with over 87% ambulatory at the one-year follow-up, compared to 78% in those treated with ORIF with locked plating.

Appleton et al. had the largest sample size of 54 distal femoral fractures treated with replacement in 52 patients [10]. The mean age was 82 years (range: 55-98 years), with 94% of the patients being female. The remaining studies included fewer patients treated with DFR (range: 12-54). The patient demographic was similar across the studies, with a majority of patients being female and the mean age of over 75 years.

In patients treated with DFR, complications included superficial and deep wound infections and peri-prosthetic fracture following further trauma, for which there were four such complications within three months of surgery [10]. The average re-operation rate among the studies was 13.3%, which was similar to 13.5% for those treated with ORIF. Information on peri-operative transfusion requirements is limited in the literature; however, according to Bettin et al., blood loss for DFR averaged 344 mL with the use of a high-thigh tourniquet [6], with only one patient requiring fluid resuscitation post-operatively.

The outcomes including length of stay and mortality are discussed and summarised in Table 1 [3,9-14]. Table 1 includes five studies that directly compared the two surgical techniques. Table 2 outlines the outcomes from three articles solely examining fracture fixation used as a surgical technique for distal femur fractures [2,15,16].

**Table 1:** Outcomes from the literature review comparing DFR with fixation. DFR: distal femoral replacement; ORIF: open reduction internal fixation; FWB: full weight-bearing

Study	Atrey et al. [9]	Hoellwarth et al. [11]		Hart et al. [3]			Tandon et al. [12]		Appleton et al. [10]	Leino et al. [13]		Ruder et al. [14]	
	DFR	DFR	ORIF	DFR	ORIF	DFR		ORIF	DFR	DFR	ORIF	DFR	ORIF
Sample number	12	53	87	10	28	21		40	54	29	39	23	35
Time to surgery (days)	5.75	2.06	1.25	-	-	-		-	-	-	-	-	-
Time to FWB (days)	3.6	-	-	-	-	1.5		77	-	-	-	-	-
Length of stay (days)	18.8	5	6	7.3	7.5	9		32	15	-	-	-	-
Mobility: ambulatory (%)	-	77.36	64.4	100	77	-		-	100	86	75	73	96
Cost	-	-	-	-	-	£10,000		£9,800	-	-	-	-	-
30-day mortality (%)	-	-	-	-	-	-		-	-	-	-	-	-
One-year mortality (%)	-	18.8	25.2	30	21.4	9.5		15	41.1	34	23	20.6	
Complications	One lymphoedema	-	-	One deep and one superficial infection, 10% required further surgery	One deep and one superficial infection, 11% required further surgery	-		-	Four periprosthetic within 3/12, one infection leading to amputation, 4.6% risk of revision at one year	31% required further surgery	23% required further surgery	8.7% required further surgery	5.7% required further surgery

**Table 2:** Outcomes from the literature review for fixation (plating) techniques. ORIF: open reduction internal fixation; FWB: full weight-bearing

Study	Smith et al. [15]	Kammerlander et al. [16]	Jennison et al. [2]
	ORIF	ORIF	ORIF
Sample number	105	43	88
Time to surgery (days)	2	1.9	2.2
Time to FWB (days)	-	-	-
Length of stay (days)	29	14.7	15
Mobility: ambulatory (%)	-	-	-
30-day mortality (%)	7	-	9.1
One-year mortality (%)	18	18.4	34.1
Complications	20/105	18.6% needed revision	9.1% required a further surgical procedure
		30.2% risk of further fragility within 3 years	
		Non-significant lower survival rate of those admitted from care home compared to own home	

Discussion

The BPT for hip fractures was introduced in April 2010 in the United Kingdom to provide optimal care for patients admitted with a neck of femur fracture by incentivising hospital trusts to meet six targets [17]. With an increasingly ageing population, such injuries are becoming much more common, with over 70,000 hip fractures reported annually throughout the country. The main aims of the BPT were to encourage prompt surgery and the appropriate involvement of geriatricians to improve patient outcomes and reduce mortality in a patient population with significant medical comorbidities [18]. The successful implementation of the BPT has led to the reduction in the average 30-day mortality from 7.1% in 2016 to 6.1% in 2019 [19].

Although less common, cases of distal femoral fractures are also increasing. These fractures are known to occur in a similar patient population as fragility hip fractures with a similar comorbidity profile. Results show the average one-year mortality for DFR is 26.6% compared to 22.1% in those treated with fixation. These mortality rates are similar to hip fractures, which range from 10% to 30% nationally, but with suboptimal care in comparison, which has led to the decision to extend the scope of the BPT to include all fractures of the femur in elderly patients. This should lead to an improvement in care for this cohort of patients, and hopefully an improvement in outcomes.

Improving outcomes can also be achieved by limiting the need for further surgery or revision, especially in patients who have significant comorbidities or score highly on the Rockwood Frailty Score [20]. Experience from a London tertiary care centre shows many patients were referred with complications related to fixation of distal femur fractures. Several failures were seen secondary to fracture malunion, chronic pain, or joint destruction and collapse after attempted plating. All of these patients were subsequently treated with a secondary procedure to remove the fixation implant and perform DFR with standard implants despite being a revision procedure. Considering the prevalence of such cases, DFR can be considered to be a primary surgical option to avoid these common complications associated with fracture fixation and those associated with the patient being non-weight-bearing.

With regard to complications, a direct comparison study observed no difference between the two groups of patients with post-operative complications; however, one in five patients in the fixation cohort developed a non-union [3], which is consistent with the literature regarding non-union rates [7]. Another study reported that, at one year, there were no complications in the DFR cohort compared to one failure of fixation, six malunions, and two infections in the fixation cohort [12]. A study examining a group of patients treated with DFR found implant-related complications in 11% or in two cases of their sample. One was secondary to infection, while the other suffered a peri-prosthetic fracture [6]. This is significantly lower than the 21.1% found to have deep infections in a study assessing complications in patients treated with DFR for various non-oncological reasons rather than just fracture [21]. There have been no documented cases of aseptic loosening of implant components, including those in a three-year follow-up study [9], which is supported by a 100% survivorship documented at four years [21].

Although the average risk of further surgery between the two treatment modalities is similar (13.3% in DFR vs. 13.5% in ORIF), the relative complications are lower with DFR; therefore, this treatment approach may provide a viable singular surgical option, especially for the cohort of patients who are frail or may find compliance with non-weight-bearing instructions difficult.

DFR involves the use of cemented stemmed tibial and femoral prostheses in a hinged construct. DFR is indicated when there is a high degree of comminution combined with intra-articular fractures within the osteoporotic bone due to the challenging nature of achieving adequate reduction and fixation [6]. Pre-existing arthritis or deformity and the need for rapid mobilisation are also significant indications for proceeding with DFR, provided the patient is fit enough from an anaesthetic perspective. A knee specialist or a trauma surgeon with appropriate experience would be needed to perform the procedure, and this can be a limiting factor in providing adequate treatment. Bettin et al. found that the average time to definite surgery was seven days for closed injuries [6], while Atrey et al. averaged 5.75 days until DFR [9]. A study comparing the use of locking plates with DFR for peri-prosthetic fractures had time to surgery for DFR averaging at 2.06 days compared to 1.25 days for ORIF [11]. With one of the primary aims of the BPT to encourage prompt surgery, this presents a limitation for the use of DFR as the primary surgical option for these fragility fractures.

The procedure allows immediate post-operative full weight-bearing compared to ORIF, where weight-bearing is traditionally restricted to allow for a union. However, surgeons are moving towards early mobilisation in their post-operative management of patients treated with ORIF using a locking plate construct and allowing protected weight-bearing or treating with an intramedullary (IM) nail. The IM device is load sharing and thus permits early weight-bearing, helping to reduce the risk of complications associated with immobility. A study comparing DFR with locked lateral plating for patients with a peri-prosthetic distal femur fracture found that all patients were fully weight-bearing in under two days following DFR, while there was an average of 11 weeks to full weight-bearing for patients post-fixation [12]. However, when directly compared for injuries around native joints, the time to discharge was 7.5 days in those treated with ORIF and 7.3 in those treated with DFR [3].

A small study comparing DFR against fixation for such fractures reported that the DFR group showed a larger proportion returning to independent walking, with quicker rehabilitation and improved knee flexion [6]. In a larger direct comparison study, all patients in the DFR group were ambulatory at one year post-operatively, although requiring the use of at least a single walking aid. In the fixation group, 23% of patients were wheelchair-bound; however, 27% of the patients were fully independent without the need for walking aids [3].

The implant cost for a DFR exceeds that of the plate and screws used for fixation. However, taking into account time until hospital discharge, the patient being fully weight-bearing, and possible community support, the overall costs of the procedures are similar, with an approximate overall cost of £10,000 for DFR and £9,800 for fixation [9,12]. Despite the slightly higher cost, the favourable outcomes with regard to weight-bearing and post-operative ambulatory status highlight an overall greater cost-benefit when using DFR compared to fixation techniques.

The relative infrequency of these fractures has led to a paucity of high-level evidence with regard to the use of DFR as a primary surgical treatment option for distal femoral fractures. Further research is required into this treatment, but it would be difficult to gather sufficient sample numbers to ensure an adequately powered randomized control trial [22]. However, the available literature points to positive outcomes when considered against the existing standard of treatment including various fixation techniques. It provides a surgical option that facilitates early mobilisation and avoids the risks associated with fixation and non-weight-bearing status. The greatest benefit may be for the cohort of patients who will not be able to tolerate a period of non-weight-bearing on the affected side or those with severely comminuted fractures with bone loss. This procedure required the expertise of an appropriately trained knee or trauma surgeon, which may pose problems in achieving early surgery and early mobilisation.

The literature provides valuable insights into the use of DFR as a treatment modality for distal femoral fractures and offers information on various treatment outcomes, particularly regarding post-operative complications and mobility status. Bettin et al. used various functional outcome scores to objectively assess patients in the post-operative period including the Knee Society Score [6]. These scores are useful to assess patient development with regard to their function; however, without a pre-operative score for a majority of patients, possibly due to trauma, a judgement of return to baseline remains a subjective opinion relying on the patient’s perspective. Therefore, the use of such scoring systems in the trauma setting is useful but should not be used in isolation to assess patient function.

Among the studies that compare DFR with fixation techniques, there is no clear algorithm for determining which is suitable for DFR. Indications for both procedures are similar, including, but not limited to, comminuted, intra-articular fractures of the distal femur. The level of comminution and radiological osteopenia suggesting poor bone stock are possible determining factors in the decision to perform DFR against fixation. Hart et al. included an inability to ambulate pre-injury as an exclusion criterion [3], whereas Bettin et al. excluded those with previous knee surgery [6]. In these studies, the level of osteoporosis was determined via subjective means of individual surgeons’ assessment of radiographs rather than aided by the use of dual-energy X-ray absorptiometry imaging. Furthermore, there is little discussion regarding the medical comorbidities of a patient including mental cognition and whether this would play a significant role in determining if DFR is appropriate. A clear description or algorithm of inclusion criteria, such as those provided by the National Institute for Health and Clinical Excellence for use of total hip arthroplasty in the treatment of acute neck of femur fractures, would be of benefit in choosing surgical candidates where DFR would be in their best interests.

Limitations

This paper has its limitations that are linked to the availability of literature on the topic. As a less prevalent injury, there is a lack of evidence directly comparing DFR with fixation, resulting in a small sample size to analyse. The heterogeneity of the studies also makes a comparison of results through meta-analysis difficult. The diversity among the studies in patient demographics and inclusion/exclusion criteria means comparison between the two treatment techniques needs to be treated with caution. Furthermore, the focus of this paper has been on comparing plating fixation techniques. Despite plating being an established method of fixation, IM nailing or even a combination of IM nailing plus plate fixation is becoming increasingly more popular in managing complex distal femoral fractures.

There is a need for further research to provide high-level evidence than currently available and investigate relative short-term mortality and long-term outcomes, which will contribute to the overall cost versus benefit profile of the procedure. A well-designed, prospective randomised control trial comparing the two treatment options, and the newer varied fixation options such as combination nailing and plating, could help establish the best practice for distal femoral fracture management. A multi-centre trial would help establish a satisfactory sample size to ensure adequate power of the study.

## Conclusions

Distal femoral fractures can be difficult to treat because of poor bone quality and pre-existing joint arthritis. Intra-articular fractures can present even greater complexity with associated morbidity and the risk of rapid traumatic arthritic progression. DFR offers an option that allows immediate weight-bearing and leaves most patients ambulatory at the long-term follow-up. Although more costly than other operative techniques, DFR avoids complications associated with fixation such as non-union and reduces the risk of further surgery through direct complications or the need for delayed arthroplasty, which is deemed more complex secondary to fixation.

With the extended BPT, to include all femoral fractures in the elderly, early mobilisation is a key step in reducing morbidity and mortality among this cohort of patients. Hence, a procedure such as DFR should be more widely considered to help achieve this. The benefits of this procedure appear to outweigh the relatively higher cost compared to plating, and, thus, DFR may have advantages within this clinical setting.
